# *Tmeff2* is expressed in differentiating oligodendrocytes but dispensable for their differentiation *in vivo*

**DOI:** 10.1038/s41598-017-00407-1

**Published:** 2017-03-23

**Authors:** Hao Huang, Peng Teng, Ruyi Mei, Aifen Yang, Zunyi Zhang, Xiaofeng Zhao, Mengsheng Qiu

**Affiliations:** 1 The College of Life Sciences, Zhejiang University, Hangzhou, 310036 China; 2 Institute of Life Sciences, College of Life and Environmental Sciences, Hangzhou Normal University, Hangzhou, 310036 China; 3 Department of Anatomical Sciences and Neurobiology, School of Medicine, University of Louisville, Louisville, KY 40292 USA

## Abstract

Myelin elaborated by oligodendrocytes (OLs) in the central nervous system (CNS) is required for saltatory conduction of action potentials along neuronal axons. We found that TMEFF2, a transmembrane protein with EGF-like and two follistatin-like domains, is selectively expressed in differentiating/myelinating OLs. Previous studies showed that TMEFF2 is capable of binding to PDGFA, which plays important roles in the proliferation, migration and differentiation of oligodendrocyte progenitor cells (OPCs). However, molecular and genetic analysis revealed that *Tmeff2* is a weak binder of PDGFA, and not required for OL differentiation and myelin gene expression *in vivo*. Together, our data suggested that *Tmeff2* is specifically upregulated in OLs, but dispensable for OL differentiation and maturation.

## Introduction

Myelin sheaths formed by OLs play crucial roles in the functioning of CNS, as they provide electrical insulation to promote conduction of axonal impulse^1^. Recent studies demonstrated that myelin sheaths provide energy and trophic support to the wrapped axons^2–4^. During embryonic development, OPCs are generated from restricted domains of neuroepithelium, such as the pMN domain in the spinal cords. Once generated, they are dispersed to occupy the entire CNS and continue to proliferate^5–7^. At later stages, OPCs especially those in the white matter regions begin to differentiate into mature OLs and form myelin sheaths^8^.

Previous studies have demonstrated that PDGFA and its receptor PDGFRA are required for OPC proliferation and prevent their differentiation at early stages^9–14^. The deficiency of PDGFRA signaling leads to premature OL differentiation, and causes severe hypomyelination at later stages due to the reduced number of OPCs^12^. On the contrary, forced expression of PDGFA *in vivo* induced hyperproliferation^9, 14^. Our recent studies have also suggested that *Nkx2*.*2* regulates the timing of OL differentiation by inhibiting the transcription of *Pdgfra* ^12, 15^. However, the delay of OL differentiation is overcome in *Nkx2*.*2* conditional mutants at later postnatal stages, suggesting the involvement of additional intracellular or extracellular factors in regulating PDGFA/PDGFRA pathway.

TMEFF2 (also known as tomoregulin and TENB2) is a transmembrane protein with EGF-like and two follistatin-like domains, but lacks an obvious functional intracellular domain^16–18^. A previous study reported that TMEFF2 specifically binds to PDGFA and suppresses fibroblast proliferation stimulated by PDGFA^19^. In this study, we found that *Tmeff2* is specifically up-regulated in OLs at the onset of cell differentiation. Given the importance of PDGFA/PDGFRA signaling in OL development, the selective expression of *Tmeff2* in differentiating OLs prompted us to hypothesize that TMEFF2 competitively binds to PDGFA and antagonizes PDGFRA signaling. However, the lack of apparent phenotypes in OL differentiation and myelin gene expression in *Tmeff2* mutant mice suggested that *Tmeff2* is dispensable for oligodendrocyte development.

## Results

### *Tmeff2* is selectively expressed in differentiating OLs

**Figure 1:**
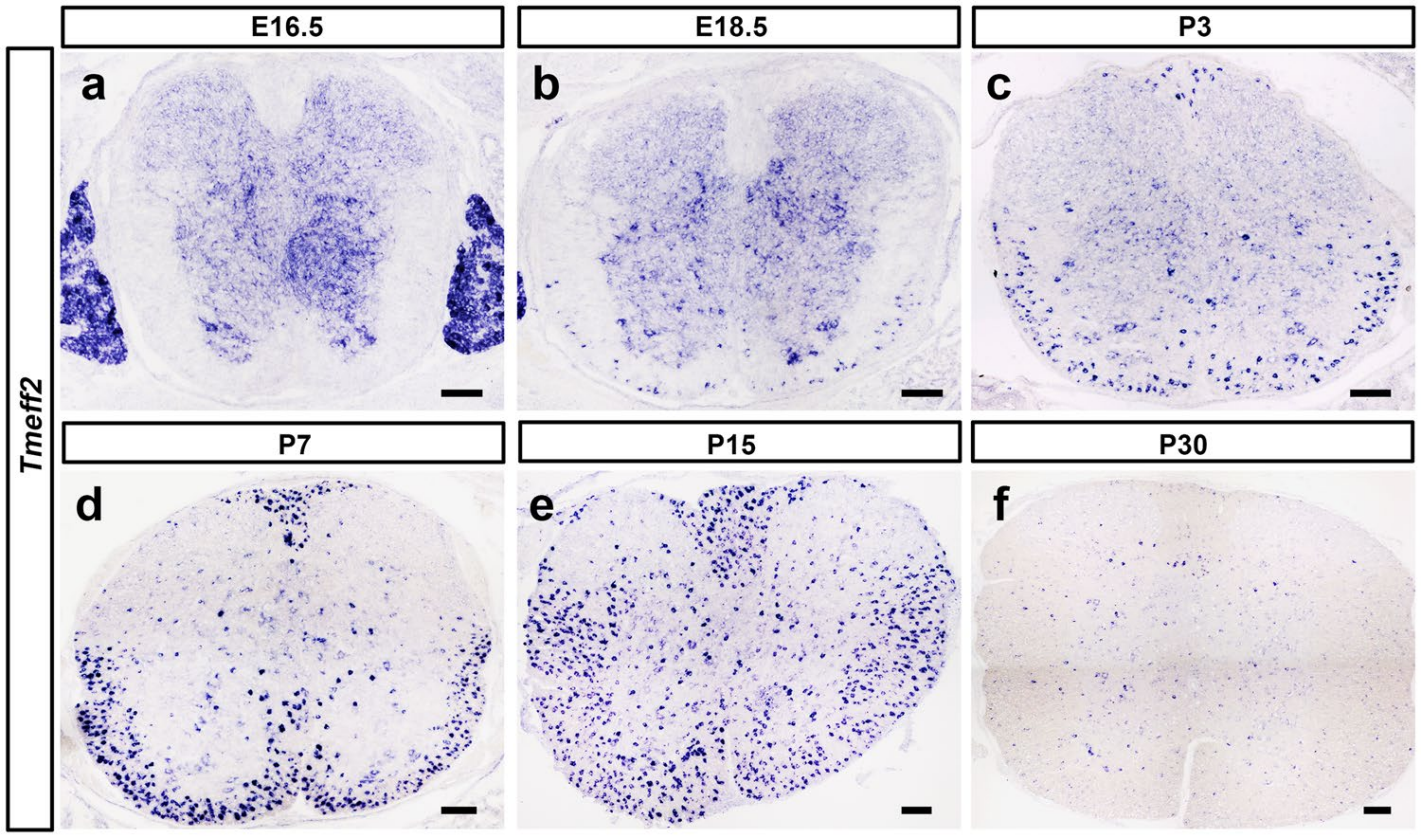
*Tmeff2* is up-regulated in differentiating OLs. (**a–f**) Sections of E16.5, E18.5, P3, P7, P15 and P30 mouse spinal cords were subjected to ISH with *Tmeff2* riboprobe. *Tmeff2* is mainly expressed in gray matter at embryonic stages, but switches its expression to OLs after birth. Bar, 100 μm.

**Figure 2:**
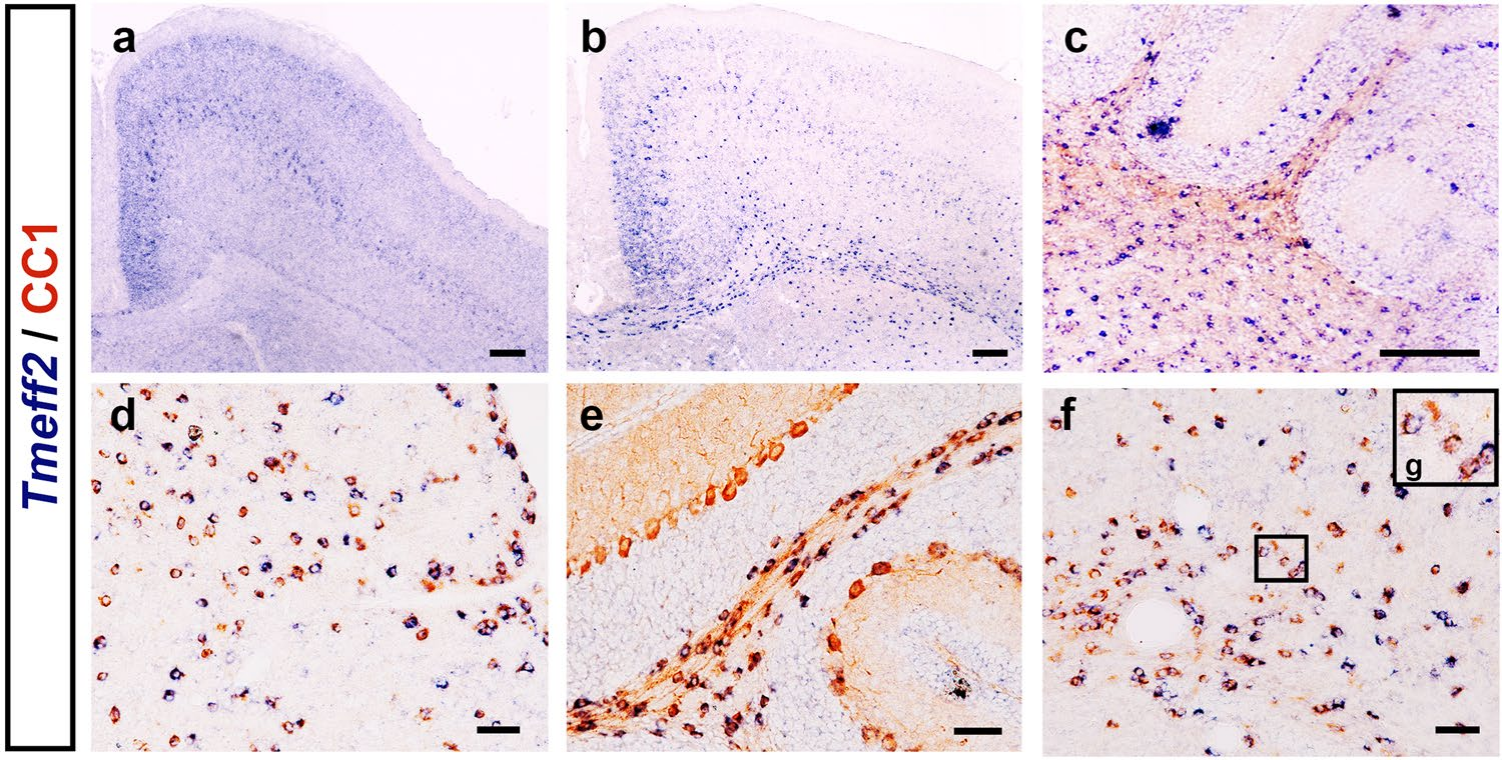
*Tmeff2*-expressing cells are immunoreactive to CC1 in different regions of the CNS. (**a–c**) *Tmeff2* expression was detected in cortex at P7 (**a**), corpus callosum (**b**) and white matter of cerebellum (**c**) at P15. Bar, 250 μm. (**d–g**) *Tmeff2* co-expresses with CC1 in spinal cords (**d**), cerebellum white matter (**e**) and corpus callosum (**f,g**) at P15. Bar, 50 μm.

To investigate whether *Tmeff2* is expressed in the developing CNS, we first examined its expression at several developmental stages by *in situ* hybridization (ISH). It was found that *Tmeff2* is primarily expressed in the gray matter of mouse spinal cords before embryonic day 18.5 (E18.5), but rapidly down-regulated after birth (Fig. 1). At the same time, *Tmeff2* expression starts to be up-regulated at E18.5 in the white matter, reaches its peak from P7 to P15, and down-regulated thereafter (Fig. 1). Similarly, in the developing cortex, *Tmeff2* transcription is first detected in cortical neurons at P7 (Fig. 2a), but later up-regulated in the white matter (corpus callosum) at P15 (Fig. 2b). In addition, *Tmeff2* is also expressed in the white matter of cerebellum (Fig. 2c). Together, these data suggested that *Tmeff2* is specifically up-regulated in cells of oligodendrocyte lineage during differentiation stage.

**Figure 3:**
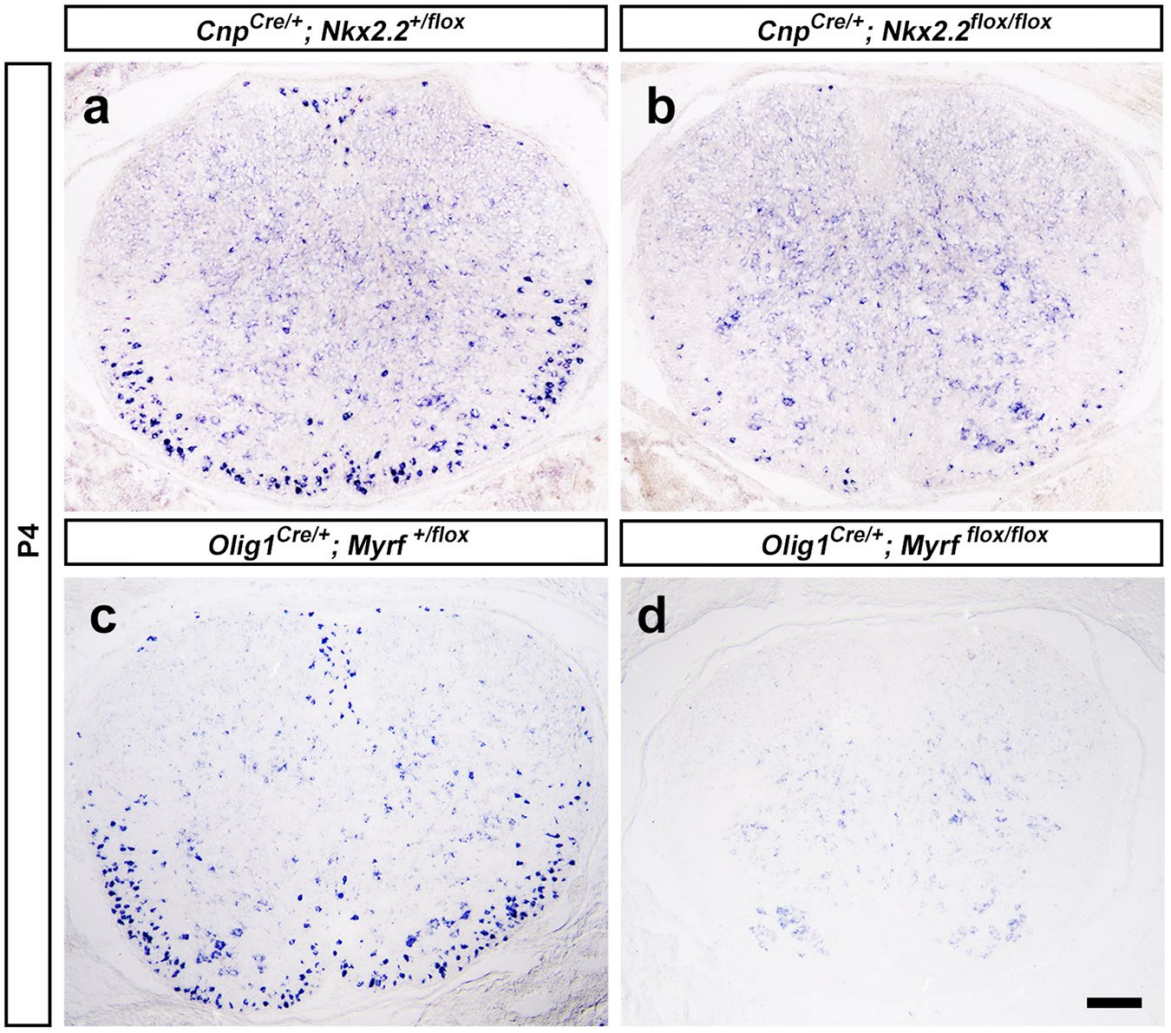
The numbers of *Tmeff2*+ cells are drastically reduced in *Nkx2*.*2* and *Myrf* CKO mice. (**a–d**) Compared with wild type mice (**a,c**), the numbers of *Tmeff2*+ cells are reduced in *Nkx2*.*2*-CKO mice (**b**) and completely lost in *Myrf*-CKO mice (**d**) at P3 stage. Bar, 100 μm.

To confirm the expression of *Tmeff2* in differentiating OLs, we carried out double staining with *Tmeff2* ISH followed by immunohistochemistry against CC1, a specific marker for differentiating/differentiated OLs^20^. As expected, all *Tmeff2*+ cells in P15 spinal cord, corpus callosum and cerebellum are CC1+ (Fig. 2d–g). In addition, we examined *Tmeff2* expression in *Nkx2*.*2* and *Myrf* conditional knock-out (CKO) mice in which OL differentiation is significantly delayed and impaired^12, 21, 22^. The results showed that the numbers of *Tmeff2*+ cells in the white matter are markedly reduced in *Nk2*.*2*-CKO mutants and completely lost in *Myrf*-CKO mutants (Fig. 3). Taken together, these results indicated that *Tmeff2* is expressed in differentiating or newly differentiated OLs in all regions of CNS.

### Disruption of *Tmeff2* in mice has no effect on OL differentiation

**Figure 4:**
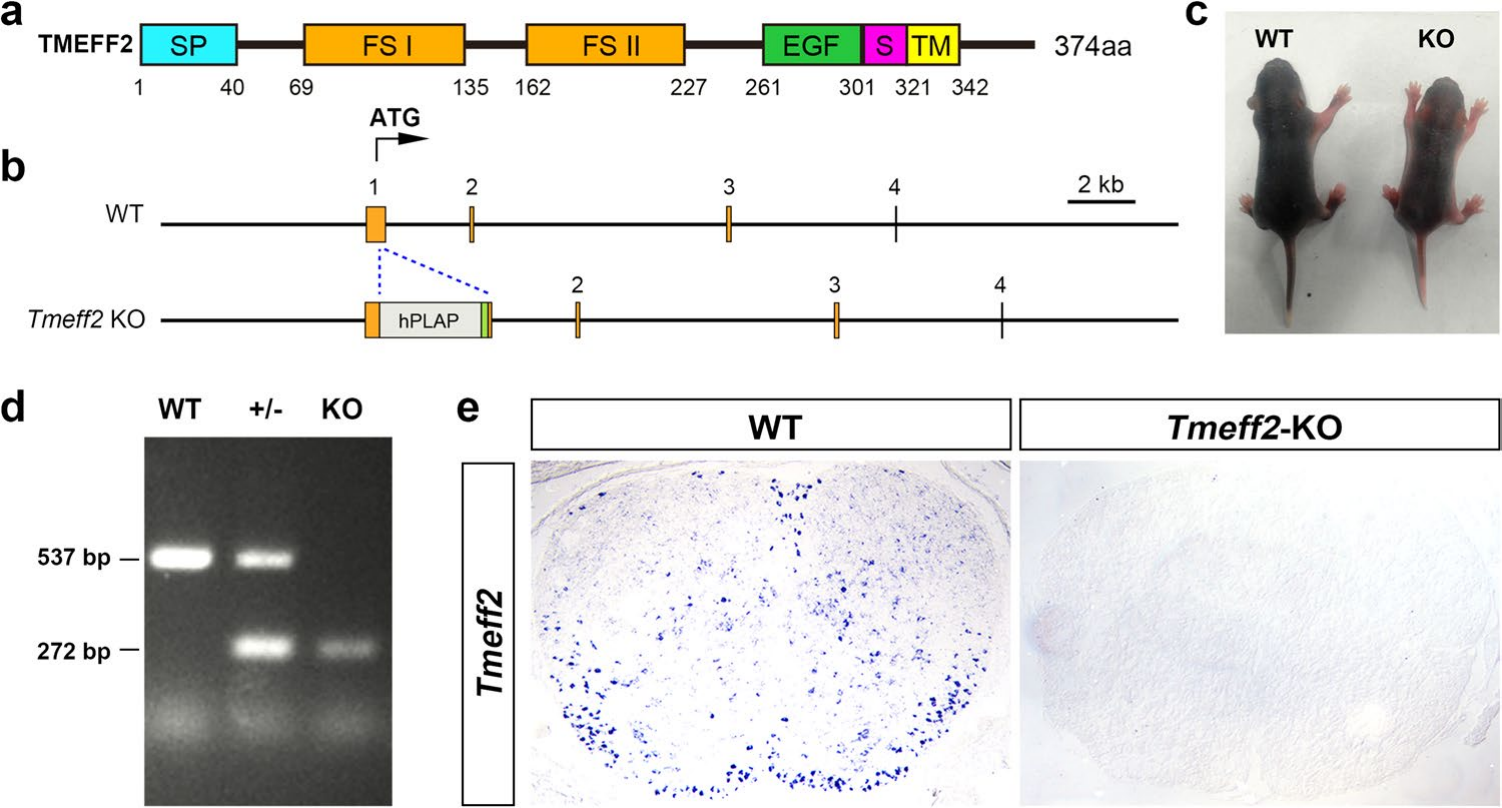
*Tmeff2* gene and *Tmeff2*-KO mice. (**a**) Structure of TMEFF2. TMEFF2 has a signal peptide (SP), two follistatin-like domains (FS I, FS II), an EGF-like domain (EGF), a shedding domain (S) and a transmembrane domain (TM). (**b**) A schematic of *Tmeff2*-KO mice. A human *PLAP* cassette was inserted after the start codon in the first exon. (**c**) The *Tmeff2*-KO mutant mice are smaller than their wild type littermates. (**d**) Genotyping results of *Tmeff2*-KO mice. (**e**) *Tmeff2* mRNA transcription could not be detected by ISH in mutant mice.

**Figure 5:**
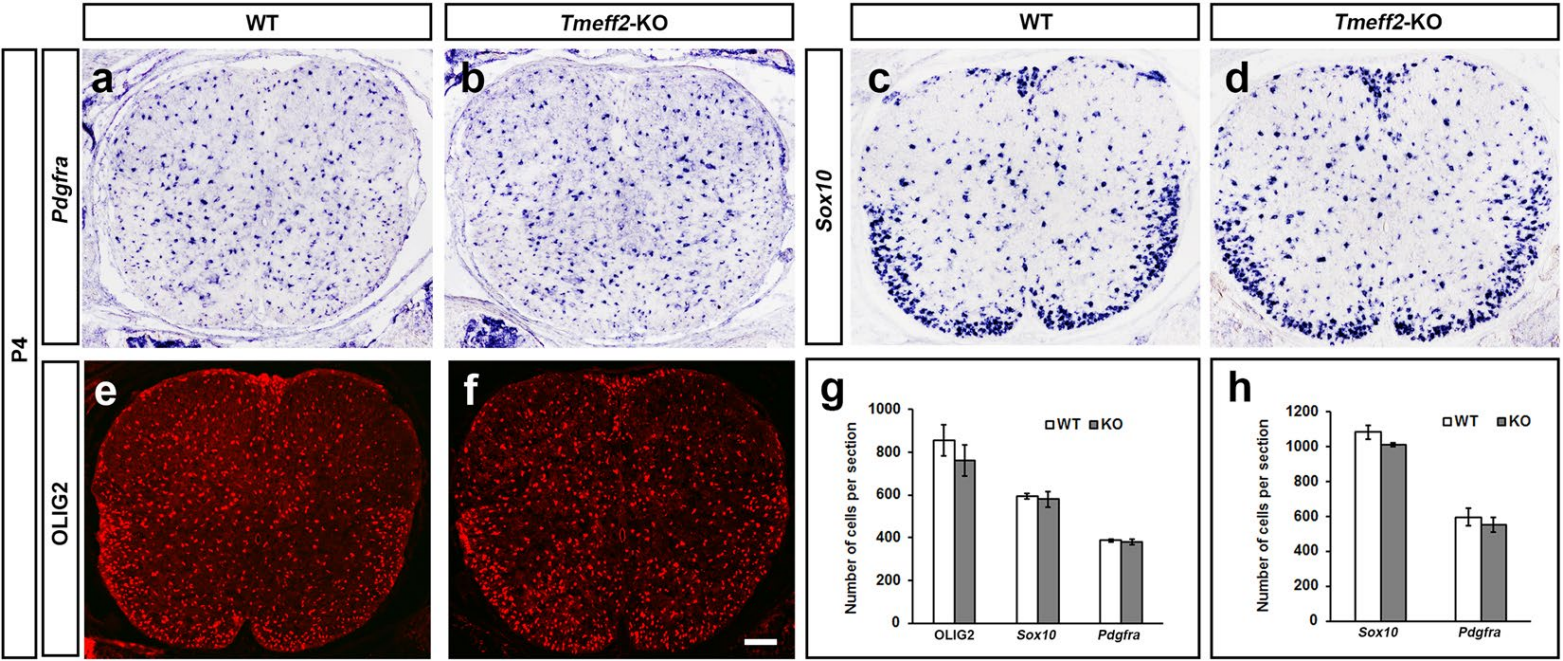
The number of OPCs is not affected in *Tmeff2*-KO mutants. (**a–f**) Expression of *Pdgfra* (**a,b**), *Sox10* (**c,d**) and OLIG2 (**e,f**) as detected by ISH or immunofluorescence in wild type and mutant spinal cords at P4. Bar, 100 μm. (**g,h**) Statistical analysis of OLIG2+, *Sox10*+ and *Pdgfra*+ OPCs in P4 (**g**) and P8 (**h**) spinal cords. n = 3, p > 0.05.

Since TMEFF2 is initially expressed in gray matter at embryonic stages (Fig. 1) and binds to PDGFA, deletion of *Tmeff2* may affect the proliferation and migration of OPCs by affecting the availability of PDGFA. This possibility was investigated by examining the expression of three OPC markers, *Pdgfra*, *Sox10* and OLIG2, in early postnatal spinal cords of *Tmeff2*-KO mutant mice. The phenotypes of mutants were confirmed by their smaller body size and lethality around weaning age^23^ (Fig. 4c), and the lack of *Tmeff2* mRNA transcription in the spinal cord tissue (Fig. 4e). Unexpectedly, there is no significant difference in the number of *Pdgfra*+ OPCs between wild type and mutant mice at P4 and P8 stages (n = 3, p > 0.05). Similarly, the numbers of OLIG2+ and *Sox10*+ cells in P4 and P8 mutants are similar to those in control tissues (n = 3, p > 0.05) (Fig. 5, and Table S1). These results indicated that *Tmeff2* is not necessary for OPC proliferation during early OL development.

**Figure 6:**
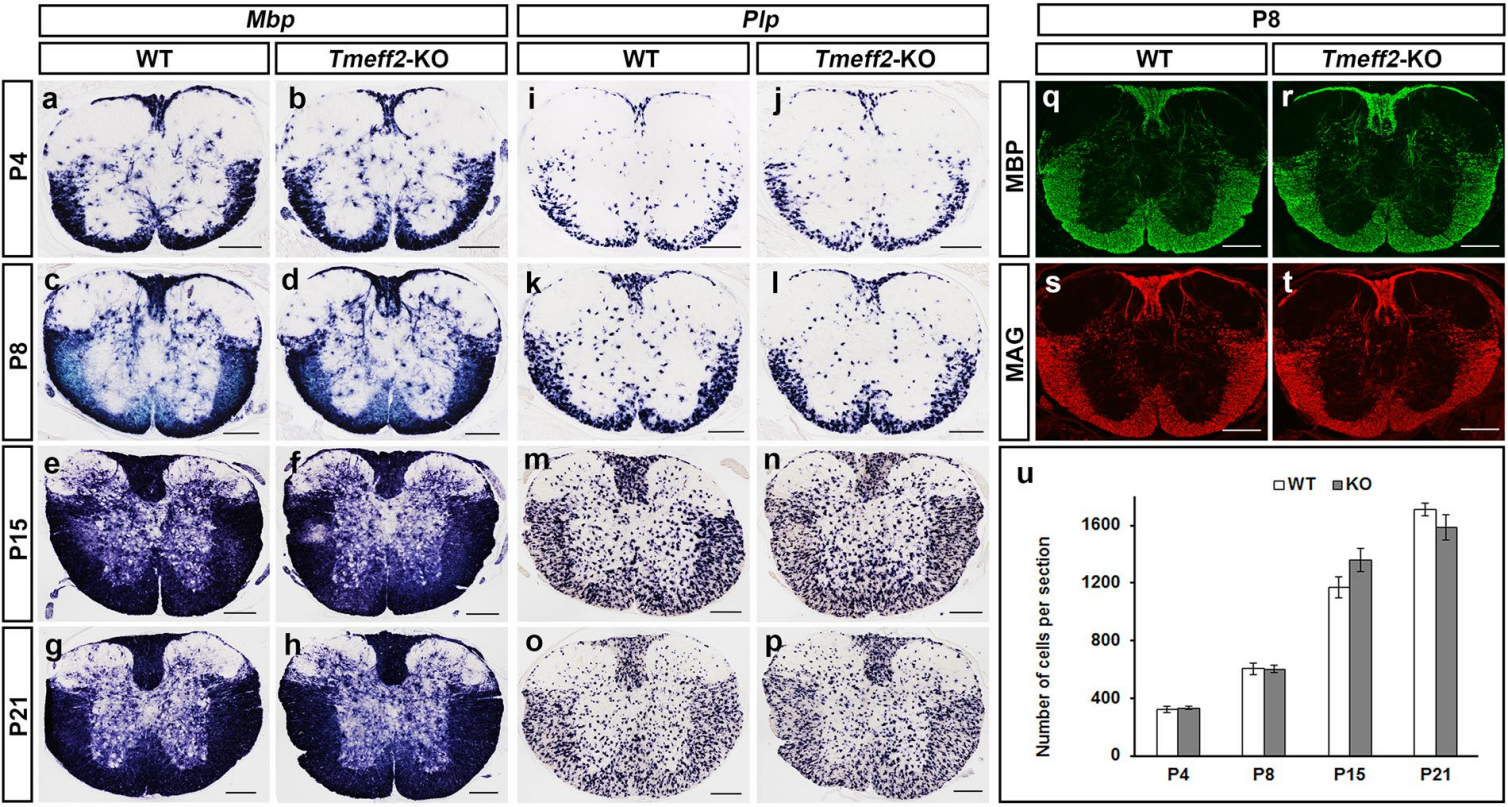
Normal differentiation of OLs in *Tmeff2*-KO mutant spinal cords. (**a–p**) Spinal cord sections of wild type and *Tmeff2*-KO mice were subjected to ISH with *Mbp* (**a–h**) and *Plp* (**i–p**) riboprobe at different stages. (**q–t**) Immunofluorescent staining with anti-MBP (**q,r**) and anti-MAG (**s–t**) in P8 wild type (**q,s**) and mutant (**r,t**) spinal tissues. Bar, 200 μm. (**k**) Statistical analysis of the number of *Plp*+ OLs in spinal cords from P4 to P21 stages. n = 3, p > 0.05.

Differentiation of OLs in *Tmeff2*-KO mutant spinal cords was examined by ISH with two mature OL markers, *Mbp* and *Plp*. To our surprise, expression of both *Mbp* and *Plp* is normal in *Tmeff2*-KO mutants at various postnatal stages (Fig. 6). As *Plp* mRNA is mainly restricted to the cell bodies and can be visualized individually, the number of *Plp*+ cells per section is counted and there is no discernible difference between control and mutant tissues (Fig. 6k and Table S2). Expression of MBP and MAG myelin proteins as detected by immunofluorescence is also similar in both genotypes (Fig. 6). These results showed that *Tmeff2* is not essential for OL differentiation in the spinal cord.

**Figure 7:**
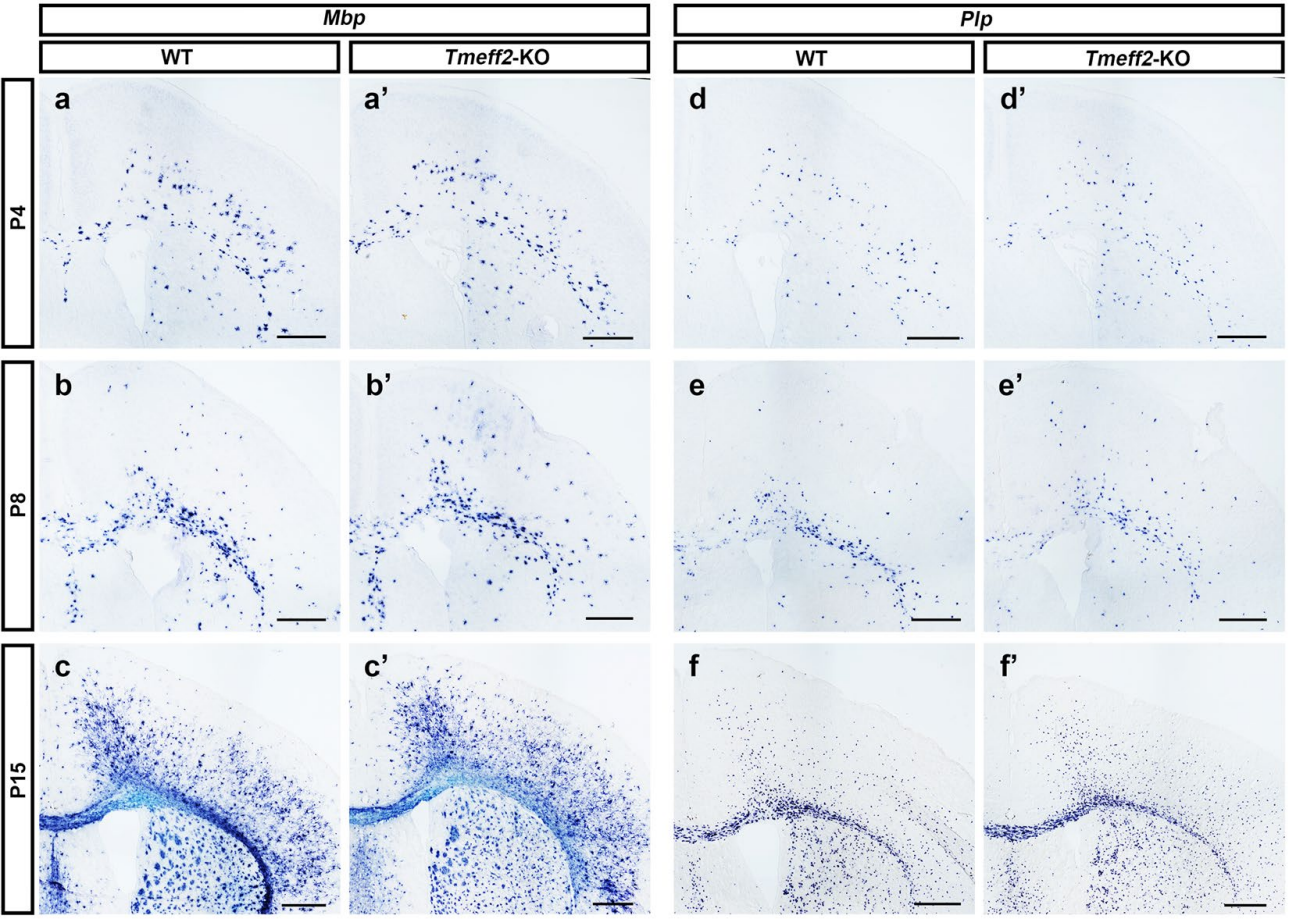
Normal differentiation of OLs in postnatal *Tmeff2*-KO mutant forebrains. (**a–f,a’–f’**) Brain sections of wild type (**a–f**) and *Tmeff2*-KO mutants (**a’–f’**) were subjected to ISH with *Mbp* (**a–c,a’–c’**) and *Plp* (**e–f,e’–f’**) riboprobe at P4, P8 and P15 stages. Bar, 500 μm.

In addition, we examined OL differentiation and myelin gene expression in mutant forebrains. As shown in Fig. 7, the timing of the onset of *Mbp* and *Plp* expression is not affected in the mutants, and their subsequent expression is also normal from P4 to P21 stages. Together, all these results indicated that *Tmeff2* is not required for the terminal differentiation of OLs in the entire CNS.

### TMEFF2 is a weak PDGFA-binding protein

Previous studies suggested that TMEFF2 regulates PDGF signaling in cancer cells by binding to PDGFA with its extracellular domain^19^. However, disruption of *Tmeff2* does not cause any alterations in OPC proliferation and differentiation (Figs 5–7), raising the question whether TMEFF2 can effectively compete with PDGFRA for binding to PDGFA.

**Figure 8:**
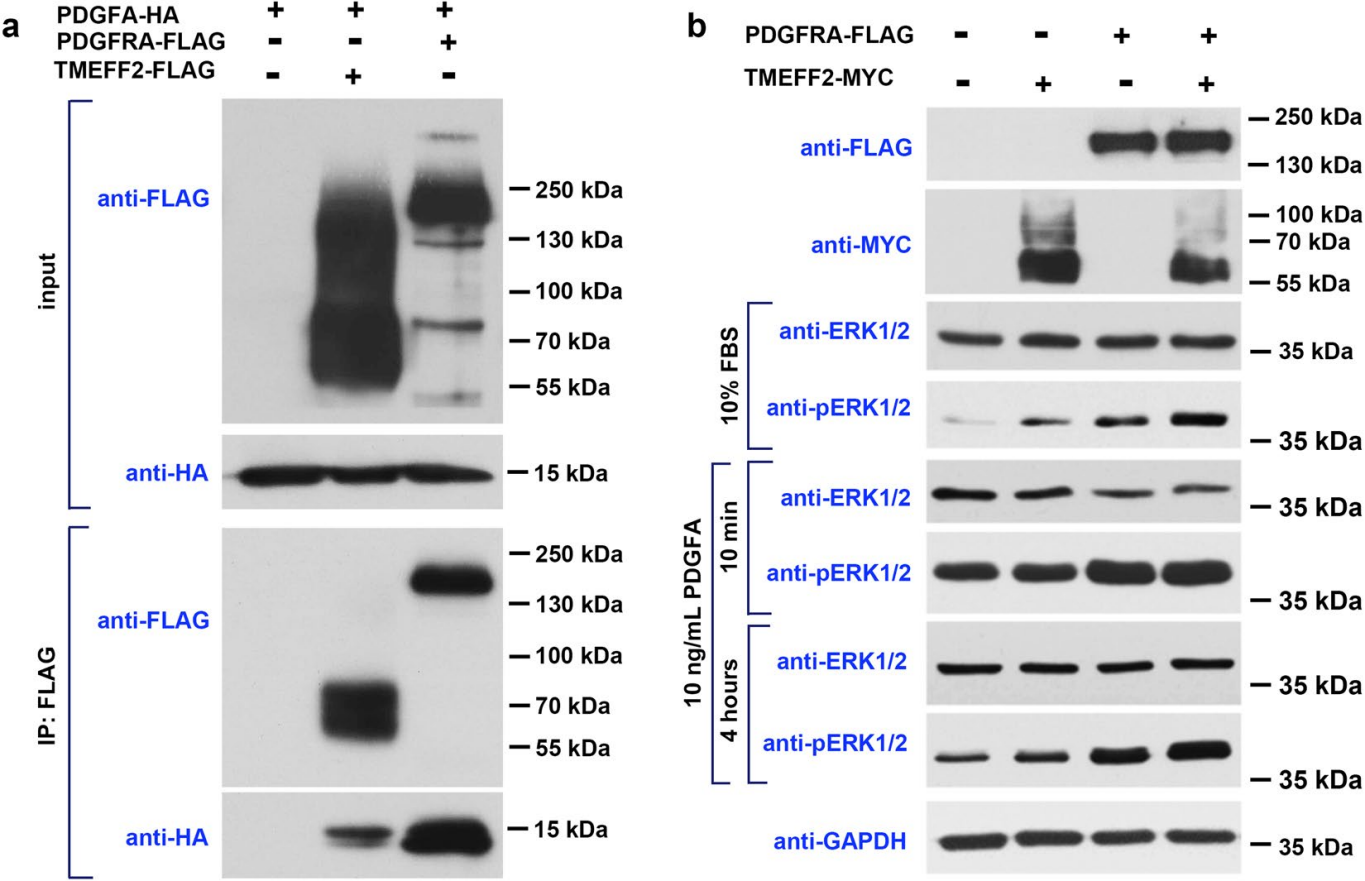
TMEFF2 is a weak PDGFA-binding protein. (**a**) Co-IP showed that the affinity between PDGFA and TMEFF2 is lower than that between PDGFA and its natural receptor PDGFRA. (**b**) TMEFF2 has a weaker effect in stimulating ERK1/2 phosphorylation, compared to PDGFRA, in DMEM media containing 10% FBS or 10 ng/mL PDGFA.

To address this question, we carried out *in vitro* protein binding assays by co-immunoprecipitation (co-IP) experiments. FLAG-tagged TMEFF2 and HA-tagged PDGFA were co-expressed in HEK293T cells, and FLAG-tagged PDGFRA served as a positive control. The co-IP results showed that TMEFF2 binds to PDGFA *in vitro* weakly, as compared to the strong binding between PDGFA and its natural receptor PDGFRA (Fig. 8a). The effects of PDGFA on PDGFRA and TMEFF2 signaling were also analyzed by the activation of pERK1/2. When 10 ng/mL PDGFA is added into the media, ERK1/2 is activated rapidly and continuously in PDGFRA overexpressing cells, but not in TMEFF2 overexpressing cells (Fig. 8b). It was previously reported that TMEFF2 acts as a receptor or co-receptor for several growth factors including PDGFA and EGF, and promotes ERK phosphorylation *in vitro* ^24^. Consistent with this finding, the level of pERK1/2 is slightly up-regulated in TMEFF2 overexpression cells, and is further increased in TMEFF2/PDGFRA co-expressing cells than in PDGFRA overexpressing cells (Fig. 8b).

Taken together, these data showed that TMEFF2 is a weak receptor/co-receptor for PDGFA-binding and ERK1/2 activation, and does not appear to function as a competitive inhibitor of PDGFRA. In this case, the lack of detectable phenotypic changes in OL differentiation in *Tmeff2*-KO mice may be due to the small effects of TMEFF2 on PDGFA binding and downstream signaling. Thus, the *in vivo* function of TMEFF2 in organ development and animal survival remains unknown.

## Discussion

Oligodendrocytes arise from specific regions of neural epithelium and subsequently migrate to the entire CNS before they differentiate and form myelin around neuronal axons. These progressive developmental events are precisely regulated by a variety of intracellular and extracellular factors^25–29^. PDGFA is a well-known extracellular factor that plays a critical role in OL development^30, 31^. The interaction between PDGFA and its receptor PDGFRA expressed in OPCs stimulates OPC proliferation and represses their differentiation until a proper cell number is achieved^9, 10^. Immediately before differentiation, *Pdgfra* is down-regulated to allow the up-regulation or activation of other critical factors, such as *Sox10* and *Myrf*, that promote terminal differentiation of OLs^21, 32^. Here, we report that TMEFF2, a type I transmembrane protein with short intracellular domain, is highly expressed by differentiating OLs (Figs 1–3). Based on the previous finding of TMEFF2 binding to PDGFA^19^ (Fig. 4), we initially hypothesized that TMEFF2 helps to promote and solidify OL differentiation by interfering with PDGFA/PDGFRA signaling. To our surprise, *Tmeff2*-KO mice do not display apparent defects in OL proliferation and differentiation (Figs 5–7). These findings have raised the possibility that TMEFF2 may not bind to PDGFA effectively and therefore does not regulate PDGFRA signaling during oligodendroglial development. Consistent with this notion, co-IP experiments showed that TMEFF2 has a much weaker binding to PDGFA than PDGFRA, and addition of PDGFA to TMEFF2 overexpressing cells only causes a weak activation of ERK1/2 (Fig. 8). These results implied that TMEFF2 may function as a low-affinity receptor/co-receptor for PDGFA, and does not appear to function as a strong inhibitor or activator of PDGFA/PDGFRA signaling under those conditions.

Although our data showed that *Tmeff2* is dispensable for OL development *in vivo*, it is not clear whether *Tmeff2* could promote OL differentiation and myelin repair after injury-induced demyelination. Considering that TMEFF2 stimulates ERK1/2 phosphorylation (Fig. 8b) and MAPK-ERK1/2 activation promotes OL differentiation and axonal myelination^33–36^, it remains plausible that *Tmeff2* can enhance OL differentiation during development and remyelination in a redundant manner. In addition, it is possible that TMEFF2 plays an unknown function in oligodendrocyte differentiation and myelin formation. Since *Tmeff2*-KO mice die before adulthood, generation of conditional mutants are necessary for dissecting out the *in vivo* function of *Tmeff2* in myelin development and repair in the future.

## Methods

### Immunohistochemistry and ISH

Tissues were fix in 4% PFA overnight at 4 °C, and transferred into 20% sucrose in PBS overnight at 4 °C. Tissues were then embedded in OCT and sectioned into 14 μm on a cryostat. Experimental procedures for immunohistochemistry and ISH were described previously^37^. For *Tmeff2* probes, 750 bp fragments corresponding to 900 nt–1650 nt of mouse *Tmeff2* mRNA (NCBI Reference Sequence: NM_019790.4) were cloned into pT7T3D-PacI vectors for transcription *in vitro*. Antibodies were used as follows: anti-OLIG2 (Millipore, AB9610, 1:1000), anti-CC1 (Abcam, ab16794, 1:500), anti-MAG (Millipore, MAB1567, 1:500), anti-MBP (Abcam, ab7349, 1:500).

### *Tmeff2*-KO mice

All animal experiments were performed in accordance with the institutional guidelines drafted by the Laboratory Animal Center, Hangzhou Normal University, and were approved by the Animal Ethics Committee of Hangzhou Normal University, China. The generation and phenotypes of *Tmeff2*-KO mice were previously described by TR Chen *et al*.^23^. The primers for genotyping are as follows: *Tmeff2*-F (5′-TCATGCTCTCCTTTGGTCGCAG-3′), *Tmeff2*-WT-R (5′-AAACATCTATGGTTCCCCACACC-3′) and *Tmeff2*-KO-R (5′-GAGCCTCATTACCTGGGATGATG-3′). Wild-type allele resulted in a 537 bp band, while mutant allele produced a 272 bp band. *Myrf* ^*flox*^ mice were obtained from Jackson Laboratory (Stock Number: 010607). Other strains such as *Nkx2*.*2* ^*flox*^ mice^38^, *Olig1-Cre* mice^39^ and *Cnp-Cre* mice^40^ were described previously.

### Construction of expression vectors and Western blot

For TMEFF2, PDGFRA and PDGFA over-expression *in vitro*, ORFs of mouse *Tmeff2* (NM_019790.4), *Pdgfra* (NM_001083316.2) and *Pdgfa* (NM_008808.3) were cloned into the pCDH-MCS-EF1-CopGFP vectors, with FLAG, MYC and HA tagged at the C-terminal. HEK293T cells in 12-well plates were transfected with these vectors and cultured in 10% FBS/DMEM or free DMEM for 24 hours. Cells in free DMEM media were treated with 10 ng/mL PDGFA for 10 min or 4 hours before lysed. All cells were then lysed in 300 μL lysis buffer containing 25 mM pH 7.4 Tris, 150 mM NaCl, 1% NP40, 0.25% sodium deoxycholate, 1 mM EDTA, protease and phosphatase inhibitors. 20 μL samples were used for western blot. Antibodies for western blot were used as follows: anti-FLAG (Sigma, F7425, 1:10000), anti-MYC (CST, 2276S, 1:10000), anti-GAPDH (Epitomics, 2251–1, 1:2000), anti-ERK1/2 (Epitomics, 1171–1, 1:2000), anti-pERK1/2 (Epitomics, 2219–1, 1:2000).

### Co-immunoprecipitation

Empty vectors and TMEFF2-FLAG, PDGFRA-FLAG expressing vectors were co-transfected with PDGFA-HA expressing vectors into HEK293T cells. 48 hours after transfection, cells in per 3.5 cm dish were treated with 1 ml lysis buffer containing 25 mM pH 7.4 Tris, 150 mM NaCl, 1% NP40, 0.25% sodium deoxycholate, 1 mM EDTA. 0.2 mL cell lysate was used for input, and 30 μL anti-FLAG Magnetic Beads (Sigma, Catalog Number M8823) were added into the left samples and incubated at 4 °C overnight. Beads were then washed with lysis buffer, and the FLAG-tagged proteins were eluted with 120 μL lysis buffer containing 10 μg 3xFlag peptide. Both input and IP samples were subjected to western bolt with anti-FLAG (Sigma, F1804, 1:10000), anti-HA (Abcam, ab9110, 1:10000).

### Statistical analysis

The experimental data were analyzed using a two-tailed unpaired Student's t-test. Statistical significance was considered to be at p < 0.05, and n = 3.

## Electronic supplementary material

Supplementary data
