# Supplementary material for: Tmeff2 is expressed in differentiating oligodendrocytes but dispensable for their differentiation in vivo
Source: Sci Rep. 2017 Mar 23;7:337. doi: 10.1038/s41598-017-00407-1 (PMC5428413; doi:10.1038/s41598-017-00407-1)
Supplement: Supplementary file 1 — Supplementary data [file 41598_2017_407_MOESM1_ESM.doc]

***Tmeff2* is expressed in differentiating oligodendrocytes but dispensable for their differentiation *in vivo***

Hao Huang^1,2,3^, Peng Teng^1,2^, Ruyi Mei^2^, Aifen Yang^2^, Zunyi Zhang^2^, Xiaofeng Zhao^2*^, Mengsheng Qiu^1,2,3,*^

^1^The College of Life Sciences, Zhejiang University, Hangzhou 310036, China.

^2^Institute of Life Sciences, College of Life and Environmental Sciences, Hangzhou Normal University, Hangzhou 310036, China.

^3^Department of Anatomical Sciences and Neurobiology, School of Medicine, University of Louisville, Louisville, KY 40292, USA.

Running Title: Expressional and functional analysis of Tmeff2 in oligodendrogenesis

^*^Correspondence and requests for materials should be addressed to M.Q. (email: [m0qiu001@yahoo.com](mailto:m0qiu001@yahoo.com) and X.Z. (email: [xiaofengzhao@yahoo.com](mailto:xiaofengzhao@yahoo.com))

**Table S1.** **The numbers of OLIG2+, *Sox10*+ and *Pdgfra*+ cells in wild type and *Tmeff2*-KO mice at P4 and P8 stages.** n=3, p>0.05.

|  | P4 | | P8 | |
| --- | --- | --- | --- | --- |
|  | WT | KO | WT | KO |
| OLIG2 | 858±72 | 762±70 | / | / |
| *Sox10* | 594±14 | 580±38 | 1082±38 | 1010±12 |
| *Pdgfra* | 388±6 | 382±12 | 596±52 | 552±42 |

**Table S2.** **The numbers of *Plp*+ cells in wild type and *Tmeff2*-KO mice at different stages.** n=3, p>0.05.

|  | P4 | P8 | P15 | P21 |
| --- | --- | --- | --- | --- |
| WT | 321±20 | 605±40 | 1360±82 | 1590±88 |
| KO | 333±8 | 604±23 | 1172±75 | 1714±43 |
